# DATABASE FOR INDICES OF AGING IN NONHUMAN PRIMATES

**DOI:** 10.1093/geroni/igz038.3472

**Published:** 2019-11-08

**Authors:** Joseph W Kemnitz

**Affiliations:** University of Wisconsin-Madison, Madison, Wisconsin, United States

## Abstract

The Primate Aging Database (PAD) is a multi-centered, relational database of biological variables in aging, captive monkeys and apes containing approximately one million data points for body weight, blood chemistry and hematology, for male and female subjects over time (https://primatedatabase.org). More than forty species are currently represented, primarily chimpanzees, macaques and common marmosets. Metadata include housing environment, social context and diet. Life history information for each species is also provided. Data in PAD is gathered from various research facilities, sanctuaries and zoos. PAD has recently been extensively revamped to enhance ease of use. Tools for data visualization and analysis in multiple formats are included. PAD has been useful for exploring biomarkers of aging in primates and for examining physiological dysregulation in aging across primate species. It also provides age-specific normative values that are valuable in clinical veterinary medicine. New data are being added to PAD, including additional subjects and variables, and additional contributors are solicited. (Supported by contract HHSN2711201800025C from the National Institute on Aging to CleMetric Data Analytics and Management, LLC.)

